# Improving the effectiveness of service delivery in the public healthcare sector: the case of ophthalmology services in Malaysia

**DOI:** 10.1186/s12913-015-1011-0

**Published:** 2015-08-28

**Authors:** Chee Yoong Foo, Ka Keat Lim, Sheamini Sivasampu, Kamilah Binti Dahian, Pik Pin Goh

**Affiliations:** All authors work in the National Clinical Research Centre, Ministry of Health, Kuala Lumpur, Malaysia

## Abstract

**Background:**

Rising demand of ophthalmology care is increasingly straining Malaysia’s public healthcare sector due to its limited human and financial resources. Improving the effectiveness of ophthalmology service delivery can promote national policy goals of population health improvement and system sustainability. This study examined the performance variation of public ophthalmology service in Malaysia, estimated the potential output gain and investigated several factors that might explain the differential performance.

**Methods:**

Data for 2011 and 2012 on 36 ophthalmology centres operating in the Ministry of Health hospitals were used in this analysis. We first consulted a panel of ophthalmology service managers to understand the production of ophthalmology services and to verify the production model. We then assessed the relative performance of these centres using Data Envelopment Analysis (DEA). Efficiency scores (ES) were decomposed into technical, scale, and congestion component. Potential increase in service output was estimated. Sensitivity analysis of model changes was performed and stability of the result was assessed using bootstrap approach. Second stage Tobit regression was conducted to determine if hospital type, availability of day services and population characteristics were related to the DEA scores.

**Results:**

In 2011, 33 % of the ophthalmology centres were found to have ES > 1 (mean ES = 1.10). Potential output gains were 10 % (SE ± 2.92), 7.4 % (SE ± 2.06), 6.9 % (SE ± 1.97) if the centres could overcome their technical, scale and congestion inefficiencies. More centres moved to the performance frontier in 2012 (mean ES = 1.07), with lower potential output gain. The model used has good stability. Robustness checks show that the DEA correctly identified low performing centres. Being in state hospital was significantly associated with better performance.

**Conclusions:**

Using DEA to benchmarking service performance of ophthalmology care could provide insights for policy makers and service managers to intuitively visualise the overall performance of resource use in an otherwise difficult to assess scenario. The considerable potential output gain estimated indicates that effort should be invested to understand what drove the performance variation and optimise them. Similar performance assessment should be undertaken for other healthcare services in the country in order to work towards a sustainable health system.

**Electronic supplementary material:**

The online version of this article (doi:10.1186/s12913-015-1011-0) contains supplementary material, which is available to authorized users.

## Background

With the ageing of the population and the increasing prevalence of chronic diseases worldwide, the demand for health services such as ophthalmology services has been escalating. In England, hospital outpatient services for ophthalmology ranked second after trauma and orthopaedics, accounting for 8.6 % of total outpatient activities [1]. In Malaysia, despite only a small increase in the proportion of elderly people in the population over the last decade (above 65 years old, 3.9 % to 5.1 % from 2000 to 2010) [2], its public hospitals have seen a two to four-fold rise in ophthalmology outpatient visits, inpatient admissions and surgeries [3]. This may in part due to the increasingly younger presentation of eye diseases [3].

Ophthalmology services in Malaysia are provided by a dual healthcare system – a tax-funded public system (primarily through the specialist hospitals operated by the Ministry of Health (MOH) and some hospitals under the Ministry of Education and Ministry of Defence) and a fee-for-service private system (through the tertiary hospitals and standalone ambulatory care centres). With the heavy subsidy in the public system, it is no surprise that the bulk of the eye diseases (70 % of total eye surgeries in 2010 [4]) are handled by the public hospitals. One negative consequence of this is the long waiting list for elective procedures. Although there is no published data, it is generally accepted by the providers that a four to six-month wait for an elective cataract surgery is the norm.

The MOH has been directing more resources to ophthalmology services to address the increasing demand. This is evident through the establishment of eight additional public ophthalmology centres between 2000 and 2012. However, given resource constraints, channelling more resources is unlikely to be sufficient by itself. The public sector needs to develop strategies to optimise its efficiency in order to achieve a sustainable healthcare system, such as a well designed benchmarking program and incentives for performance [5, 6].

The study was initiated after the MOH Ophthalmology Service Management Working Committee (OSMWC - *“Ahli Jawatankuasa Kerja Pengurusan Perkhidmatan Oftalmologi”* in Malay) approached the authors to discuss possible ways to benchmark the performance of ophthalmology centres under its wings. They were also interested in discovering any weakness of their current service delivery to maximise the use of resources. The committee was made up of senior ophthalmologists from various MOH Hospitals.

### Performance Benchmarking in Health Services

Three common approaches to benchmarking health services performance discussed in modern literature are (1) ratio based measures (e.g. severity adjusted average length of stay), (2) stochastic frontier analysis (SFA) and (3) data envelopment analysis (DEA) [7]; each with its own advantages and disadvantages. The ratio based approach while being simple, is often less desirable due to its inadequacy in capturing the multiple dimensions of health service inputs and outputs [7]. On the other hand, SFA differentiates true inefficiency from random observation error but it requires making difficult-to-test assumptions on the production relationship between the inputs and outputs. In contrast, the non-parametric DEA does not assume any relationship, but attributes all deviations from the performance frontier as inefficiency [8]. Furthermore, DEA also features the ability to derive various indicators of performance and to identify peers most relevant to each unit for mutual learning. Examples of its use include efficiency assessment of: hospitals [9, 10], health programmes [11], and dialysis centres [12].

In this study, we took advantage of the DEA approach to develop a performance benchmarking model for the MOH ophthalmology service. Specifically, our objectives were: (1) to benchmark the service performance among all MOH ophthalmology centres in Malaysia and assess the performance variations; (2) to demonstrate the potential output gains achievable if all centres were able to arrive at the performance frontier based on the DEA model; and (3) to test if certain environmental and organisational variables were related to the performance scores observed.

## Methods

We analysed ophthalmology centres located within the MOH hospitals for 2011 and 2012. There were a total of 36 centres in 2011. These centres are located within a minor specialist hospital (≤10 specialty or sub-speciality services), a major specialist hospital (≤20 specialty or sub-speciality services) or a state hospital (>21 specialty or sub-speciality services). A new centre was established in 2012, but was excluded from the analysis to allow comparison over both years. The 36 ophthalmology centres included in the analysis are hereafter referred to as the “decision making units” (DMUs).

### Data sources

Data were obtained from the National Healthcare Establishment and Workforce Survey (NHEWS Hospital), National Eye Database (NED) Monthly Census and National Cataract Registry (NCR). NHEWS Hospital is an annual hospital facility survey collecting data on healthcare services, activities and workforce conducted by Clinical Research Centre of MOH. NED Monthly Census and NCR gather facility-level and patient-level data respectively on ophthalmology services outputs and outcomes within the MOH hospitals. Details on methodology of these databases are published and accessible publicly [13, 14]. Table 1 lists all variables used in this study and their respective sources.

**Table 1:** Variables used in this study and their respective sources

Variables		Data source
Input		
	Number of operating room	NHEWS
	Total elective operative hour (per 4-week month)	NHEWS
	Number of full time ophthalmologist	NHEWS
	Number of assistance medical officer	NHEWS
	Number of nurses	NHEWS
	Number of operating microscope	NHEWS
	Number of phacoemulsifier	NHEWS
	Number of vitrectomy devices	NHEWS
Output		
	Total number of cataract surgery	NHEWS
	Total number of glaucoma surgery	NHEWS
	Total number of vitreo-retinal surgery	NHEWS
	Total number of corneal surgery	NED Monthly Census
	Total number of oculaplasty surgery	NED Monthly Census
	Total number of outpatient cases	NED Monthly Census
	Total number of inpatient cases	NED Monthly Census
	Percentage of patients with post-operative visual acuity of 6/12 or better within 3 months following cataract surgery	NED CSR
	Percentage of patients without infectious endophthalmitis post-cataract surgery	NED CSR
Environmental factors		
	Availability of day surgery services	NHEWS
	Hospital Type	NHEWS
	Total population of the district within which the hospital is located	DOS
	Proportion of local population above 60 years old	DOS

*NHEWS* National Healthcare Establishment and Workforce Survey, *CSR* National Eye Database Cataract Surgery Registry, *NED* National Eye Database, *DOS* Department of Statistics, Malaysia

### Model building

The building of the DEA model required an understanding of the ophthalmology service production. We first consulted two experienced practising MOH ophthalmologists (including author PPG) to identify the key input and output variables from the data sources (Table 1). We constructed a reference model based on their contribution and subsequently varied the input and output combinations based on the literature to produce five alternative models (Table 2). Basic DEA analysis was conducted on all models to observe the effect different variables had on the DEA results (i.e. percentage of frontier DMUs and the mean technical efficiency score (ES)). We then organised a meeting with the OSMWC to verify the variables and to select the most appropriate model according to their knowledge of the ophthalmology service production and the model performance in terms of their sensitivity to model changes. Analyses were then carried out using the determined model.

**Table 2:** Combination of inputs and outputs used in various DEA models

	DEA models					
Variable names	1	2	3	4	5	Ref^a^
Elective operating hours	√	√	√	√	√	√
Permanent ophthalmologist	√	√	√	√	√	√
Supporting clinical staff	√	√	√		√	√
Assistant Medical Officers				√		
Nurses				√		
Operative microscope	√		√	√	√	√
Phacoemulsifier	√		√	√	√	√
Vitrectomy device	√		√	√	√	√
Total number of input	6	3	6	7	6	6
Total surgeries^b^			√			
Cataract surgery	√					
Quality-adjusted cataract surgery^c^		√		√	√	√
Non-cataract surgery	√	√		√		√
Glaucoma surgery					√	
Vitreo-retinal surgery					√	
Corneal surgery					√	
Oculaplasty surgery					√	
Outpatient visits	√	√	√	√	√	√
Inpatient admissions	√	√	√	√	√	√
Total number of output	4	4	3	4	7	4
Total number of variables	10	7	9	11	13	10

^a^Reference model built based on initial discussion with two MOH ophthalmologists

^b^Sum of total quality-adjusted cataract surgeries and non-cataract surgeries

^c^Total number of cataract surgeries multiplied by two quality indicators : (1)Percentage of cases achieving visual acuity of 6/12 or better within 3 months following cataract surgery and (2) Percentage of cases without post-operative endophthalmitis

### Data envelopment analysis

As DEA is sensitive to outliers, we first checked all outlying variables and found no indications of reporting errors or missing data.

DEA analysis can adopt either an input or output perspective under either variable return to scale (VRS) or constant return to scale (CRS) assumptions. An input-oriented analysis can be used to explore the extent to which resource can be reduced while still maintaining the same level of output; and output-oriented analysis addresses the question of how more outputs can be delivered given the existing resources [15]. For this study, we took the output perspective because the DMUs have little control over their inputs – labour employment and purchase of equipment; these are under the purview of the MOH central administration. Our analysis made the VRS assumption that the scale of production varied according to level of input.

The main outcome measure of the study was the VRS technical ES, which reflects the room for potential efficiency improvements arising from currently ineffective service delivery processes. In addition, we also derived the scale and congestion ES. Scale ES informs the likely optimal sizes of DMUs for best productivity gain whereas congestion ES shows the efficiency level taking into account that some outputs might be undesirable (such as complication of surgeries), that minimising such outputs could improve efficiency. The technical explanation of each efficiency score is available in Additional file 1.

All output oriented ES are interpreted similarly: A DMU has an ES of 1.0 if it lies on the performance frontier; higher than 1.0 if it is below the frontier. In the latter case, the DMU is benchmarked against the best performing centre(s) most similar to itself (the ‘peers’). To illustrate, an ES of 1.5 indicates that the DMU could potentially have produced 50 % additional outputs with its existing input levels. Using this interpretation, we estimated the potentially achievable output gains in all three aspects of efficiency performance assuming all DMUs were able to achieve levels of performance close to the frontier.

### Robustness checks

To ascertain the robustness of the analysis, we have also undertaken two robustness checks. First, we performed a series of sensitivity assessments to the changes of input and output variables before we met the OSMWC. This exercise allowed us to examine variables that could affect our result and thus the conclusions. A second assessment was done using a bootstrapped DEA approach (of 2000 resampling cycles) in order to ascertain the robustness of the results given random sampling variations [16].

### Second stage regression analysis

To explore whether different environmental and organisational conditions can systematically affect the variation of the efficiency scores, we undertook a second stage Tobit regression analysis [16].The bias-corrected technical ES from bootstrapped DEA was regressed against a series of independent factors. These factors are listed on Tables 1 and 3. A *p* ≤ .05 was considered statistically significant.

**Table 3:** Descriptive statistics of the input, output and environmental variables

Variables	2011		2012	
	Med	IQR	Med	IQR
Input				
Operating Room	1.0	1.0	1.0	1.0
Elective operating hours	80.0	97.0	80.0	81.0
Permanent ophthalmologist	3.0	4.0	4.0	4.0
Supporting clinical staff	4.0	1.0	4.0	2.0
Assistant Medical Officers	4.0	4.8	5.0	6.0
Nurses	8.0	7.5	8.5	10.0
Operative microscope	2.0	1.0	2.0	2.0
Phacoemulsifier	2.0	1.0	2.0	1.3
Vitrectomy device	1.0	1.0	1.0	2.0
Output				
Total surgeries^a^	723.1	864.9	805.4	928.7
Cataract surgery	725.5	683.0	848.0	835.5*
Quality-adjusted cataract surgery^b^	679.6	648.9	774.4	773.7*
Non-cataract surgery	43.5	216.0	31.0	155.0
Glaucoma surgery	13.5	29.8	10.5	27.8
Vitreo-retinal surgery	0.0	75.5	0.0	76.0
Corneal surgery	0.0	0.0	0.0	0.0
Oculoplasty surgery	0.0	0.0	0.0	1.0
Outpatient visits	19722.0	15254.0	21319.0	14658.0
Inpatient admissions	955.0	1263.0	908.0	969.0
Environmental factors				
Proportion of centre with day surgery service (%)	81 %	-	89 %	-
Hospital type (by proportion (%))		-		
Major Specialist Hospital	52.8 %	-	52.8 %	-
Minor Specialist Hospital	8.3 %	-	8.3 %	-
State Hospital	38.9 %	-	38.9 %	-
	Mean	SD	Mean	SD
Proportion of population above 60 years old (within the centre's district)(%)^c^	-	-	7.3 %	3.1 %
Total population of the district ('000)^c^	-	-	511.3	397.6

*p-value < 0.05 by Wilcoxon signed rank test

*Med* Median, *IQR* Interquartile range, *SD* Standard deviation

^a^Total numbers of quality-adjusted cataract surgeries and non-cataract surgeries

^b^Total number of cataract surgeries adjusted with two quality indicators: (1)Percentage of cases achieving visual acuity of 6/12 or better within 3 months following cataract surgery and (2) percentage of post-operative endophthalmitis

^c^Data available for 2012 only

The DEA and regression analyses were performed using R version 3.1.1. [17] Two R packages were used: the Benchmarking package [18] and the FEAR package [19].

## Results

### Descriptive statistics

Medians and inter-quartile ranges (IQR) are shown in Table 3 for each input and output variable used in the various DEA models. Only the number of cataract surgeries and quality-adjusted cataract surgeries were significantly different (*p* < 0.05) between both years. The details of inputs and outputs for each individual DMU are shown in Additional file 2.

### Performance benchmarking

**Fig. 1:**
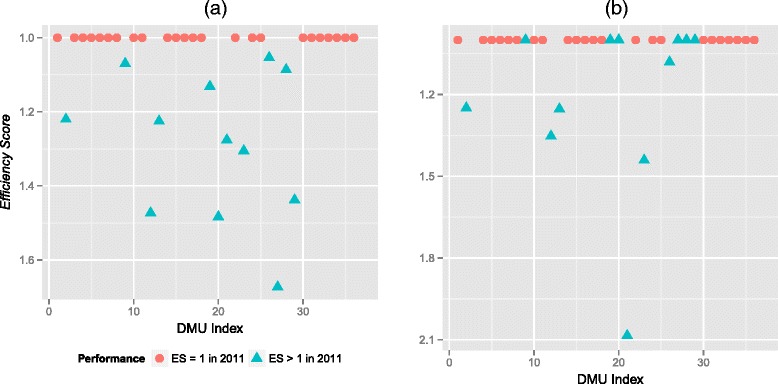
**a** Performance variation of DMUs in 2011. **b** Performance variation of DMUs in 2012. A DMU has an efficiency score (ES) of 1.0 if it lies on the performance frontier; higher than 1.0 if it is below the frontier.

Figure 1 shows the variation of the DMUs’ technical ES across two years. A noticeably higher number of DMUs appear on the performance frontier (ES = 1) in 2012 compared to 2011. Out of 12 DMUs with ES > 1 in 2011, six moved to the frontier the following year. In contrast, only one out of the twenty-four DMUs deteriorated from the performance frontier in 2012. Nevertheless, the mean technical ES improved only slightly from 1.10 (SD 0.18, range 1.00 – 1.70) to 1.07 (SD 0.20; range 1.00 – 2.09). The detail of technical, scale and congestion ES for each DMU are available in Additional files 3 and 4.

### Potential output gain

**Fig. 2:**
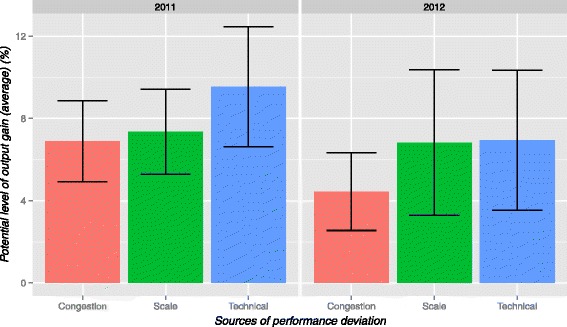
Potential output gain attributable to various sources of performance deviation for 2011 & 2012. Error bars show 95 % confident interval.

The potential output gains could be derived from the mean technical, scale and congestion ES. As shown in Fig. 2, the overall output of the MOH Ophthalmology services in 2011 could potential be increased by 10 % (SE ± 2.92) by improving the service delivery processes (technical efficiency).If the right size were achieved (scale efficiency), output could potentially be increased by a further 7.4 % (SE ± 2.06). In addition, 6.9 % (SE ± 1.97) of observed lost output was due to the unwanted outcomes (congestion efficiency). The potential output gain reduced in 2012 (6.9 ± SE 1.97, 6.8 ± SE 3.5, 4.4 ± SE 1.9 for technical, scale and congestion efficiency respectively) as some DMUs moved to the performance frontier.

### Regression analysis

Only hospital type appeared to have a significant explanatory on the DEA efficiency score (Table 4). A DMU located in a state hospital was found to be associated with lower ES (better performance) than those located in major and minor specialist hospitals.

**Table 4:** The effect of environmental factors on the DEA technical efficiency scores^+^

Environmental factors		Coefficient	Standard error
Availability of day surgery services			
	No	-	
	Yes	0.07	0.06
Hospital Type^a^			
	Major Specialist Hospital	-	
	Minor Specialist Hospital	−0.08	0.07
	State Hospital	−0.10	0.05*
Proportion (%) of population above 60 years old (within the centre's district )		−1.79 x 10^−3^	0.00
Total population of the district ('000)		1.11 x 10^−4^	0.00

^+^dependent variable is the bootstrap DEA efficiency score computed using output-oriented VRS model of 2000 resampling

*p-values < 0.05; *n* = 36 for both year; other coefficients failed to reach statistical significance

^a^Hospital type is used as a proxy to indicate the scope of clinical service available within the attached hospital of the ophthalmology centre

All variables above were test simultaneously controlling for year dummy

Constant not shown for brevity

### Robustness check

**Fig. 3:**
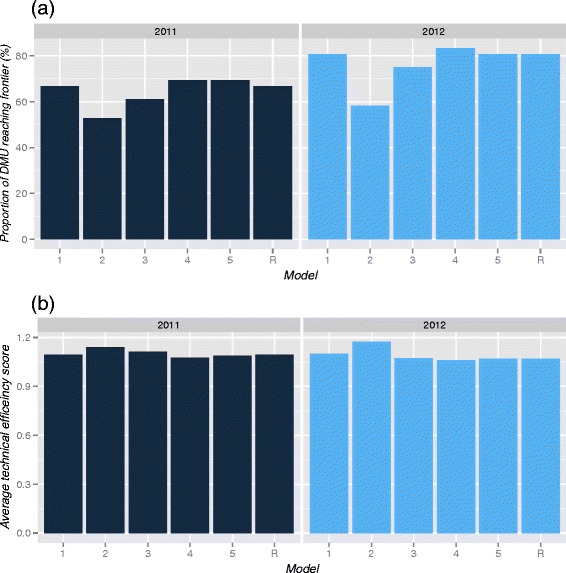
**a** The effect of model changes on the proportion of DMU reaching frontier. **b** The effect of model changes on the mean technical efficiency score. All are variable return to scale models.

The reference model and alternative models 1, 4 and 5 produced similar results in both 2011 and 2012 (Fig. 3). However, the OSMWC rejected the alternative models on the following grounds: the output was not quality adjusted (Model 1), the two categories of clinical supporting staff were substitutable and could be grouped together (Model 4). Meanwhile, the OSMWC suggested that including five individual non-cataract surgeries (Model 5) would result in an unfair benchmark because they were produced in small numbers by a few DMUs.

Model 2 and 3 gave a smaller proportion of efficient DMUs on the performance frontier compared to the other four models. Model 2 was constructed using surgical devices as an indicator of the mean of capital stock. Moreover, the OSMWC also considered that these devices were essential resources and the rate limiting factors in the production of eye surgeries. Not taking surgical devices into account would provide an unfair assessment for DMUs with limited surgical equipment. Similarly, as cataract surgeries made up the bulk of the workload in all DMUs, the OSMWC concurred that it should be distinguished from non-cataract surgeries (Model 3). Consequently, the OSMWC chose the reference model as the final model to reflect the production of their ophthalmology services.

The bootstrapped DEA generated a bias-corrected estimate of ES based on the final model. This approach identified a consistent list of low performing DMUs as the standard ES (100 % match for 2011 and 86 % match for 2012). Details of the analysis are available in Additional file 5.

## Discussion

We found that one-third of the centres may have performed sub-optimally (technical ES > 1) in 2011, which, if optimised could potentially have delivered considerably greater outputs without requiring additional resource investment. This includes a potential 10 % technical efficiency gain (i.e. by improving the service delivery mechanism), a 7 % potential scale efficiency gain (if they operate at the right scale) and a likely 7 % congestion efficiency gain (if surgical complications were minimised). The relative performance improved in 2012, with a lower potential output gain. DMUs located in state hospitals were associated with better performance.

Findings of this study may affect several policy considerations relevant to ophthalmology services. First, using DEA to condense information across multiple dimensions of service input and output in ophthalmology care has the potential to contribute to designing an effective benchmarking program. The intuitive DEA score can help service managers and policy makers visualise the system performance and examine the potential impact of ineffective resource use [20]. For example, the OSMWC were able to reflect on the outcomes and provide qualitative insights into the possible reasons for certain sub-performing centres after visualising the DEA result. A lack of leadership succession in one of the DMUs and insufficient surgical equipment in another were among the observations.

Our active engagement with the stakeholder throughout the research process was a major strength of the study and adds credibility to the analysis. This level of engagement may facilitate policy makers and managers to adopt the findings and to take actions against known causes of poor performance. Indeed, the OSMWC has already expressed interest in incorporating such an analysis in their regular management meetings to monitor their own performance.

Secondly, the analysis suggested that the MOH ophthalmology service could produce higher outputs with the existing capacity. The important next step would be detailed diagnostic studies to help explain the likely causes of the performance differentials. Are there inefficient work processes? Should we up- or down- scale certain sub-performing centres? Are poor patient outcomes a cause of the inefficient resource use? Policy makers could then make decisions about which strategies to adopt for promoting efficient behaviours, for example, changing the structure of organisation, increasing the DMU’s scale size and changing the service process [21].

Being located in a hospital with a wider scope of clinical services (proxied by hospital type) was the only significant variable explaining the ES variation [22]. However, some important confounders likely to be correlated with the ES were not able to be controlled due to the lack of data as well as the small sample size. The broader implications of the findings, therefore, may need to be interpreted with cautions. In contrast to our analysis, the existing literature shows that day surgery produces greater efficiency performance [23, 24]. The small sample size might not have allowed us to detect the often small difference in term in efficiency improvement between DMUs with and without day surgery services. Alternatively, the level of performance of day surgery services in Malaysia at that moment may not yet be able to deliver a significant efficiency differential [25].

Although some compromises were made in specifying the inputs and outputs of the benchmarking DEA model, the results were generally robust. Sensitivity analysis showed that quality adjustment had little effect on efficiency, probably because the variation in quality among the DMUs was small. There is evidence that case-mix adjustment results in small differences if the samples are homogeneous [26], which could be the case for this study because all DMUs were operating in tertiary care settings under MOH central administration. Some studies also considered inpatient beds [9, 27] and financial capital [12, 27] as inputs, but these two variables were not available to us. The labour inputs should also ideally be constructed in terms of staff full-time equivalence (FTE) or working hours which take into account part-time workers rather than number of full-time staff. However, as only a minority of MOH institutions hire part-time staff, the OSMWC decided that this was the best possible model given the available data.

Several key limitations of DEA should be considered when interpreting the findings. DEA is a non-parametric efficiency analysis that depends heavily on the accuracy of the data used, and it assumes the right level of inputs and outputs for each centre are captured [16, 20]. However, data quality is never perfect and the result must be interpreted with a good level of knowledge about the quality of the data used. Secondly, because the exact level and scope of input and output in healthcare services can never been determined with certainty, variation of service performance derived by these efficiency analyses may sometimes suffer because the resources consumed and outputs delivered were not fully captured. For example, we have learnt from the local service managers that there may be variation in the number of workforce inputs throughout the year due to their redistribution or some un-captured outputs such as ad-hoc preventive eye services offered. Minimising these errors could improve the reliability of the benchmarking results; for example, by capturing the full-time equivalent number of the workforce as input resources instead of using the absolute head count, and by developing a robust case-mix system. While these methods are a promising way of improving the reliability of DEA benchmarking, they are largely non-existent in lower resource settings such as Malaysia.

Finally, one should bear in mind that DEA efficiency scores are relative measures. Improvement in ES from 2011 to 2012 does not necessarily indicate real efficiency improvement because deterioration in the performance of peers would produce similar results. Similarly, receiving an ES = 1.0 does not necessarily mean that the DMUs have no further opportunities for efficiency gains [28]. DMUs lying on the frontier should always explore the potential for greater efficiency.

## Conclusions

Using DEA for benchmarking service performance of ophthalmology care could provide insights for policy makers and service managers into the overall performance in an otherwise difficult to assess scenario (due to the multi-dimensional input–output nature of healthcare services) [20]. The considerable potential output gains estimated indicates that effort should be invested into understanding what drove the performance variation and optimise the resource use. Similar performance assessment should be undertaken for other healthcare services in the country in order to work towards a sustainable healthcare system.

## Additional files

Additional file 1:
**Data Envelopment Analysis (DEA) technical details.** (PDF 182 kb) Additional file 2:
**Inputs and outputs data for 2011 & 2012.** (PDF 310 kb) Additional file 3:
**DEA efficiency scores of all models by DMU and index year.** (PDF 157 kb) Additional file 4:
**Scale type, scale and congestion efficiency scores by DMU and index year.** (PDF 125 kb) Additional file 5:
**VRS technical efficiency scores under standard DEA and bootstrap DEA with their respective rankings.** (PDF 48 kb)

## Abbreviations

DEA: Data Envelopment Analysis
ES: Efficiency scores
MOH: Ministry of Health
OSMWC: Ophthalmology Service Management Working Committee
SFA: Stochastic frontier analysis
DMU: Decision making unit
NHEWS: National Healthcare Establishment and Workforce Survey
NED: National Eye Database
NCR: National Cataract Registry
VRS: Variable return to scale
CRS: Constant return to scale
IQR: Inter-quartile ranges
SD: Standard deviation
SE: Standard error
FET: Full-time equivalence
