# Supplementary material for: Improving the effectiveness of service delivery in the public healthcare sector: the case of ophthalmology services in Malaysia
Source: BMC Health Serv Res. 2015 Aug 28;15:349. doi: 10.1186/s12913-015-1011-0 (PMC4551382; doi:10.1186/s12913-015-1011-0)
Supplement: Additional file 1: — Data Envelopment Analysis (DEA) technical details. (PDF 182 kb) [file 12913_2015_1011_MOESM1_ESM.pdf]

Additional file 1

Data Envelopment Analysis (DEA) technical details

Following seminal work by Farrell [1] the concept of technical efficiency was developed and could be studied from two perspectives. From an input perspective, an organisation is technically efficient if output is produced with the least possible amount of input. This perspective explores the question of how many inputs can be reduced while maintaining the same level of output. From the output perspective, an organisation is considered technically efficient if it achieves the maximum output mix given their current inputs. In this case, it addresses the question of how much more outputs be produced given the existing inputs level.[2] This study took on the output perspective as individual hospital of the public sector in Malaysia have little control over their inputs level especially in term of labour and equipment. These input elements are often under the purview of the Ministry's central administration.

The organisation or unit being examined in a DEA study is called the decision making unit (DMU). DEA measures technical efficiency by first identifying a "best practice" production possibility frontier based on those DMUs achieving highest output mix for their inputs levels. These DMUs lying on the frontier would be considered efficient compared to others in the sample and would be assigned a score of 1.0. Other DMUs would then be benchmarked against these frontier centres most similar to themselves (the 'peers') and then be given scores of more than 1.0 by comparing their output / input ratio. For instance, an efficiency score of 1.5 indicates that the DMU could have produced 50% additional outputs with their existing input levels if compared to those at the frontier.

The model is summarised below:

$$TE_c = Max\ \frac{\sum_r U_r y_{rc}}{\sum_i V_i x_{ic}}$$

Subjected to $\frac{\sum_r U_r y_{rc}}{\sum_i V_i x_{ic}} \leq 1;\ \forall h$ and $U_r, V_i \geq 0;\ \forall r, \forall i$

Where

TE_c = technical efficiency score given to DMU $c\ (c = 1 \dots \dots a), c$ = number of DMU

U_r = weight given to output $r(r = 1 \dots \dots s)$, s = number of outputs

V_i= weight given to input i $(i = 1 \dots \dots t)$, t = number of inputs

y_{rc}= amount of output r produced by DMU c

x_{ic}= amount of input i produced by DMU c

Technical efficiency can be computed under constant returns to scale (CRS) and variable returns to scale (VRS) assumptions. The former assumed there was no productivity gain as the size of ophthalmology centre increased whereas the latter assumed there were economies of scale and changes in inputs would lead to disproportionate changes in outputs. Linear programming is used to maximize the TE score by finding the optimal input (V_i) and output weights (U_r) according to the model above.

Decomposition of DEA Scores

We then decomposed the CRS technical efficiency score into three components: VRS technical efficiency, scale efficiency and congestion efficiency. The conceptual relationship between the various sources of efficiency is demonstrated by the formula below. [3]

$$CRS\ technical\ efficiency = Scale\ efficiency \times Congestion\ efficiency \times VRS\ technical\ efficiency$$

Scale efficiency assumes a hypothetical optimal scale of operations i.e. if the DMU was too big or too small, changes in scale could lower cost and improve efficiency. This is given by:

$$SE_c = \frac{TE_c(y_c, x_c|CRS)}{TE_c(y_c, x_c|VRS)} = \frac{TE_{c;\,CRS}}{TE_{c;\,VRS}}$$

Comparing the efficiency score in CRS model to that of VRS model gives us the scale efficiency score. The further the departure from a score of 1, the higher the potential lost from not having right scale.

Both CRS and VRS technical efficiency scores assume strong disposability of outputs (SDO), i.e. all outputs produced during the production of ophthalmology services were desirable. In contrast, congestion efficiency assumes weak disposability of outputs (WDO), i.e. it takes into account that some outputs from ophthalmology services might not be desirable, for

instance complications of surgeries. It is defined as the ratio of VRS technical efficiency under SDO (TE(VRS,S)) to the technical efficiency measured under WDO (TE(VRS,W)), illustrated by the following mathematical notation [4]:

$$CE_c = \frac{TE_c(y_c, x_c|VRS, S)}{TE_c(y_c, x_c|VRS, W)} = \frac{TE_{c;\,VRS,S}}{TE_{c;\,VRS,W}}$$

Therefore, the VRS technical efficiency score represents the “pure” technical efficiency that reflects the deviation of performance from those achieved by the frontier centres.

Reference

1. Farrell MJ: **The Measurement of Productive Efficiency**. *J R Stat Soc Ser A* 1957, **120**:pp. 253–290.

2. Scales B: *Data Envelopment Analysis: A Technique for Measuring the Efficiency of Government Service Delivery*. Canberra: Steering Committee for the Review of Commonwealth/State Service 1997; 1997:1–142.

3. Valdmanis VG, Rosko MD, Mutter RL: **Hospital quality, efficiency, and input slack differentials**. *Health Serv Res* 2008, **43**:1830–1848.

4. Fare R, Grosskopf S, Lovell CAK: *Production Frontiers*. Cambridge University Press; 1994:296.
