# Supplementary material for: Improving the effectiveness of service delivery in the public healthcare sector: the case of ophthalmology services in Malaysia
Source: BMC Health Serv Res. 2015 Aug 28;15:349. doi: 10.1186/s12913-015-1011-0 (PMC4551382; doi:10.1186/s12913-015-1011-0)
Supplement: Additional file 2: — Inputs and outputs data for 2011 & 2012. (PDF 310 kb) [file 12913_2015_1011_MOESM2_ESM.pdf]

Additional file 2 Input and Output data (2011 & 2012)

DMU	Year 2011															
	Input levels							Output levels								
Index	x2	x3	x5	x6	x7	x8	x9	y1	y2	y3	y4	y5	y6	y7	y8	y9
1	64	1	0	3	1	1	0	311	20	0	0	0	3695	488	0.01	0.0000
2	80	3	4	3	4	2	0	738	24	0	0	0	11394	166	0.06	0.0000
3	52	2	3	5	2	2	1	553	0	0	0	0	17694	329	0.09	0.0000
4	77	3	2	4	1	1	0	891	6	0	0	0	24500	1576	0.07	0.0000
5	42	1	3	4	2	1	0	318	0	0	0	0	12346	625	0.08	0.0000
6	48	1	1	1	1	0	1	75	3	0	0	0	5303	208	0.06	0.0000
7	64	2	1	2	1	1	0	237	63	0	0	0	4277	209	0.09	0.0000
8	320	15	11	16	3	2	2	1980	84	108	41	65	53203	2350	0.09	0.0000
9	128	7	7	27	2	2	1	1630	42	67	48	58	33815	1963	0.09	0.0025
10	65	3	3	2	1	2	1	713	11	0	0	0	19896	848	0.13	0.0000
11	150	6	4	25	2	2	1	2648	0	347	0	0	54582	1923	0.06	0.0000
12	72	4	1	4	1	2	0	325	0	0	0	0	13295	49	0.03	0.0000
13	160	7	6	23	4	2	1	1577	21	74	0	0	25018	1858	0.12	0.0023
14	90	6	5	10	3	3	2	911	28	195	0	0	28075	1533	0.10	0.0000
15	298	22	7	11	4	4	3	1813	83	1295	0	159	43362	1871	0.09	0.0005
16	180	8	4	7	2	2	0	673	0	0	0	287	19548	2795	0.10	0.0000
17	56	1	3	3	1	1	0	435	2	0	0	0	11977	642	0.06	0.0000
18	72	2	3	1	0	1	0	505	0	0	0	0	12984	907	0.22	0.0000
19	108	4	5	5	1	1	0	811	1	0	0	0	17353	1059	0.06	0.0000
20	77	3	4	4	2	2	1	674	0	0	0	0	15803	1003	0.07	0.0000
21	48	2	3	4	2	1	0	285	0	0	0	0	11307	585	0	0.0000

22	192	6	4	10	4	3	2	1156	36	349	0	10	40705	1987	0.09	0.0000
23	128	6	5	6	2	1	1	778	27	70	0	0	23076	839	0.06	0.0000
24	164	7	3	6	2	2	0	450	4	0	365	0	20251	472	0.08	0.0000
25	74	3	5	3	2	2	0	953	1	0	0	0	24810	1357	0.01	0.0000
26	80	2	3	2	2	2	1	691	1	0	0	0	18581	899	0.04	0.0000
27	320	8	7	6	2	4	2	557	19	59	0	0	23641	1446	0.03	0.0000
28	128	5	7	5	3	4	1	1112	16	80	21	18	31617	1473	0.05	0.0000
29	79	3	2	3	1	2	1	536	0	0	0	0	13498	753	0.07	0.0000
30	56	3	3	4	1	2	2	409	24	0	0	0	10717	2200	0.04	0.0000
31	148	5	6	4	2	1	1	2036	0	166	0	0	29659	2057	0.06	0.0000
32	64	2	2	4	2	1	0	787	19	0	0	0	15196	119	0.06	0.0000
33	480	3	5	5	2	2	0	2006	63	0	0	0	42581	2683	0.06	0.0019
34	220	5	4	24	1	2	1	1229	35	328	156	82	27006	685	0.12	0.0008
35	280	8	5	14	2	3	2	1978	84	452	149	0	38482	2898	0.08	0.0010
36	64	1	3	1	1	0	0	623	63	0	0	0	14670	856	0.05	0.0000
Average	131.3	4.7	4.0	7.3	1.9	1.8	0.8	927.9	21.7	99.7	21.7	18.9	22608.8	1214.2	0.10	0.0

	Year 2012															
DMU	Input levels							Output levels								
Index	x2	x3	x5	x6	x7	x8	x9	y1	y2	y3	y4	y5	y6	y7	y8	y9
1	64	1	1	3	1	1	0	388	20	0	0	0	3750	417	0.01	0.0025
2	80	4	4	4	4	2	1	901	25	0	0	0	11465	214	0.03	0
3	52	2	3	5	2	2	1	518	0	0	0	0	18725	369	0.07	0.0016
4	112	3	2	3	2	1	0	1035	8	0	0	0	26218	1120	0.04	0
5	42	1	3	4	2	1	0	327	4	0	0	0	12845	449	0.11	0
6	20	1	1	2	1	2	1	71	3	0	0	0	4941	198	0.06	0

7	54	2	1	3	1	1	0	309	16	0	0	1	6524	180	0.08	0
8	186	17	11	16	3	2	2	2594	160	174	55	44	40443	2257	0.08	0
9	117	7	6	9	2	2	1	1493	19	63	27	21	36120	1367	0.09	0
10	72	3	3	2	1	2	1	769	14	0	0	0	8940	325	0.13	0
11	168	6	6	28	2	4	5	2155	32	0	0	10	48352	897	0.05	0
12	64	4	2	5	1	1	0	369	0	0	0	0	10454	58	0.03	0
13	168	7	6	9	3	5	2	970	8	97	0	0	26464	2254	0.04	0
14	80	5	5	10	2	3	2	1302	21	170	0	0	25060	1507	0.07	0
15	298	24	7	26	4	4	3	1772	50	1187	0	167	46422	1891	0.12	0
16	144	8	4	7	2	2	0	722	0	0	0	356	22498	924	0.12	0
17	56	2	2	3	1	1	0	472	2	0	0	0	12947	615	0.04	0
18	64	2	3	1	1	1	0	673	9	0	0	0	11368	561	0.06	0
19	121	4	6	16	1	1	0	845	3	0	0	0	21834	1045	0.07	0.0036
20	77	3	4	5	1	1	1	860	0	0	0	0	18369	1018	0.08	0
21	64	4	3	4	2	2	1	298	0	0	0	0	8715	365	0.01	0
22	148	5	4	9	3	3	2	1310	38	438	0	57	44795	2019	0.1	0.0016
23	128	6	5	6	2	2	1	834	9	54	0	0	22198	972	0.08	0.0026
24	92	6	4	6	3	2	0	521	4	0	312	0	24310	518	0.09	0
25	50	3	5	2	2	2	0	1118	7	0	0	0	24972	830	0	0.0018
26	80	2	3	2	2	1	0	672	0	0	0	0	17517	780	0.04	0.0015
27	144	6	7	6	3	3	2	851	30	69	0	0	20803	5132	0.04	0
28	144	7	5	5	3	3	1	1730	12	4	36	36	32380	1711	0.07	0
29	74	3	2	3	1	2	1	673	0	0	0	0	13837	630	0.06	0
30	48	2	4	4	1	2	2	64	13	0	2	1	11043	1373	0.01	0
31	192	5	5	6	3	2	1	1760	0	148	0	0	24264	1377	0.05	0
32	74	2	1	3	2	1	0	1031	32	5	0	0	18225	105	0.06	0

33	480	8	5	15	3	3	1	3139	75	365	0	0	53974	2685	0.07	0.0007
34	220	6	6	26	1	2	2	1644	61	337	332	129	29241	734	0.1	0
35	320	6	5	12	3	4	2	1836	73	359	213	0	36965	2911	0.1	0
36	60	1	4	1	1	1	0	724	63	0	0	0	13725	918	0.14	0
Average	121.0	4.9	4.1	7.5	2.0	2.1	1.0	1020.8	22.5	96.4	27.1	22.8	22519.5	1131.3	0.1	0.0004

x2 - total elective operative hour; x3 - Number of full time ophthalmologist; x5 - Number of assistance medical officer; x6 - Number of nurses; x7 - Number of operating microscope; Number of phacoemulsifier; x9 - Number of vitrectomy devices; y1 - Total number cataract surgery; y2 - Total number glaucoma surgery; y3 - Total number vitreo-retinal surgery; y4 - Total number corneal surgery; y5 - Total number oculoplasty surgery; y -Total number of outpatient cases; y7 - Total number of inpatient cases; y8 - proportion of patients with post-operative visual acuity worse than 6/12 within 3 months following cataract surgery; y9 - infectious endophthalmitis post-cataract surgery per thousand cases
