# Supplementary material for: Improving the effectiveness of service delivery in the public healthcare sector: the case of ophthalmology services in Malaysia
Source: BMC Health Serv Res. 2015 Aug 28;15:349. doi: 10.1186/s12913-015-1011-0 (PMC4551382; doi:10.1186/s12913-015-1011-0)
Supplement: Additional file 3: — DEA efficiency scores of all models by DMU and index year. (PDF 157 kb) [file 12913_2015_1011_MOESM3_ESM.pdf]

Additional file 3 DEA efficiency scores of all models by DMU and index year

Year 2011

DMU Index	DC	Efficiency Score (VRS, Output-Oriented Model)						Efficiency Score (CRS, Output-Oriented Model)					
		Ref[1]	1	2	3	4	5	Ref[1]	1	2	3	4	5
1	1	1.0	1.0	1.1	1.0	1.0	1.0	1.47	1.47	1.47	1.47	1.00	1.46
2	1	1.2	1.2	1.4	1.3	1.2	1.2	1.29	1.28	1.56	1.29	1.29	1.29
3	1	1.0	1.0	1.0	1.0	1.0	1.0	1.01	1.01	1.01	1.01	1.00	1.01
4	1	1.0	1.0	1.0	1.0	1.0	1.0	1.00	1.00	1.00	1.00	1.00	1.00
5	0	1.0	1.0	1.0	1.0	1.0	1.0	1.00	1.00	1.00	1.00	1.00	1.00
6	0	1.0	1.0	1.0	1.0	1.0	1.0	1.38	1.38	1.56	1.38	1.00	1.38
7	1	1.0	1.0	1.1	1.0	1.0	1.0	1.35	1.33	1.95	1.81	1.23	1.00
8	1	1.0	1.0	1.0	1.0	1.0	1.0	1.25	1.25	1.67	1.31	1.25	1.12
9	1	1.1	1.1	1.1	1.1	1.1	1.0	1.09	1.09	1.20	1.16	1.09	1.00
10	0	1.0	1.0	1.0	1.0	1.0	1.0	1.02	1.02	1.02	1.03	1.00	1.01
11	1	1.0	1.0	1.0	1.0	1.0	1.0	1.00	1.00	1.00	1.00	1.00	1.00
12	1	1.5	1.5	1.5	1.5	1.1	1.5	1.54	1.54	1.54	1.54	1.06	1.54
13	1	1.2	1.2	1.2	1.3	1.2	1.2	1.50	1.44	1.65	1.50	1.50	1.49
14	1	1.0	1.0	1.0	1.1	1.0	1.0	1.00	1.00	1.00	1.08	1.00	1.00
15	1	1.0	1.0	1.0	1.0	1.0	1.0	1.00	1.00	1.00	1.18	1.00	1.00
16	1	1.0	1.0	1.0	1.0	1.0	1.0	1.00	1.00	1.01	1.04	1.00	1.00
17	1	1.0	1.0	1.2	1.0	1.0	1.0	1.12	1.12	1.15	1.12	1.12	1.12
18	0	1.0	1.0	1.0	1.0	1.0	1.0	1.00	1.00	1.19	1.00	1.00	1.00
19	1	1.1	1.1	1.7	1.1	1.1	1.1	1.20	1.25	1.76	1.24	1.20	1.20
20	1	1.5	1.4	1.5	1.5	1.5	1.5	1.49	1.47	1.49	1.52	1.48	1.49
21	0	1.3	1.3	1.3	1.3	1.3	1.3	1.39	1.39	1.44	1.39	1.39	1.39
22	1	1.0	1.0	1.0	1.0	1.0	1.0	1.02	1.02	1.02	1.36	1.00	1.00
23	1	1.3	1.3	1.4	1.3	1.3	1.3	1.52	1.52	1.61	1.60	1.50	1.49

24	1	1.0	1.0	1.3	1.4	1.0	1.0	1.00	1.00	1.31	1.77	1.00	1.00
25	1	1.0	1.0	1.0	1.0	1.0	1.0	1.00	1.00	1.00	1.00	1.00	1.00
26	0	1.1	1.1	1.1	1.1	1.0	1.1	1.06	1.06	1.06	1.06	1.04	1.06
27	1	1.7	1.7	1.7	1.7	1.6	1.7	2.08	2.08	2.16	2.21	2.05	2.07
28	1	1.1	1.1	1.1	1.1	1.0	1.0	1.17	1.17	1.17	1.30	1.13	1.11
29	1	1.4	1.4	1.4	1.5	1.4	1.4	1.47	1.48	1.47	1.49	1.47	1.47
30	1	1.0	1.0	1.0	1.0	1.0	1.0	1.00	1.00	1.00	1.00	1.00	1.00
31	1	1.0	1.0	1.0	1.0	1.0	1.0	1.00	1.00	1.00	1.00	1.00	1.00
32	1	1.0	1.0	1.0	1.0	1.0	1.0	1.00	1.00	1.08	1.00	1.00	1.00
33	1	1.0	1.0	1.0	1.0	1.0	1.0	1.00	1.00	1.00	1.00	1.00	1.00
34	1	1.0	1.0	1.0	1.0	1.0	1.0	1.00	1.00	1.00	1.07	1.00	1.00
35	1	1.0	1.0	1.0	1.0	1.0	1.0	1.00	1.00	1.00	1.11	1.00	1.00
36	0	1.00	1.00	1.00	1.00	1.00	1.00	1.00	1.00	1.00	1.00	1.00	1.00
Average		1.10	1.09	1.14	1.11	1.08	1.09	1.18	1.18	1.27	1.25	1.13	1.16

[1]Reference model built based on initial discussion with two MOH ophthalmologists

DC – centre with day surgery services

		Year 2012											
DMU		Efficiency Score (VRS, Output-Oriented Model)						Efficiency Score (CRS, Output-Oriented Model)					
Index	DC	Ref[1]	1	2	3	4	5	Ref[1]	1	2	3	4	5
1	0	1.00	1.00	1.34	1.00	1.00	1.00	1.81	1.64	2.15	1.81	1.00	1.81
2	1	1.25	1.42	1.25	1.32	1.20	1.25	1.26	1.57	1.26	1.33	1.22	1.26
3	1	1.04	1.04	1.04	1.04	1.00	1.04	1.05	1.05	1.06	1.05	1.00	1.05
4	1	1.00	1.00	1.00	1.00	1.00	1.00	1.00	1.00	1.00	1.00	1.00	1.00
5	1	1.00	1.00	1.00	1.00	1.00	1.00	1.00	1.00	1.00	1.00	1.00	1.00
6	1	1.00	1.00	1.00	1.00	1.00	1.00	1.42	1.73	1.42	1.44	1.33	1.42
7	1	1.00	1.00	2.02	1.00	1.00	1.00	1.94	2.05	2.15	1.95	1.70	1.93
8	1	1.00	1.00	1.00	1.00	1.00	1.00	1.00	1.00	1.01	1.00	1.00	1.00
9	1	1.00	1.00	1.00	1.00	1.00	1.00	1.00	1.00	1.00	1.00	1.00	1.00
10	1	1.00	1.00	1.35	1.03	1.00	1.00	1.00	1.00	1.36	1.03	1.00	1.00
11	0	1.00	1.00	1.00	1.00	1.00	1.00	1.00	1.00	1.00	1.00	1.00	1.00
12	1	1.35	1.36	1.80	1.36	1.24	1.35	1.35	1.43	1.85	1.37	1.29	1.35
13	1	1.25	1.39	1.25	1.27	1.25	1.25	1.43	1.44	1.46	1.43	1.38	1.37
14	1	1.00	1.00	1.00	1.00	1.00	1.00	1.00	1.00	1.00	1.00	1.00	1.00
15	1	1.00	1.00	1.00	1.00	1.00	1.00	1.00	1.00	1.00	1.21	1.00	1.00
16	0	1.00	1.00	1.08	1.01	1.00	1.00	1.00	1.00	1.14	1.24	1.00	1.00
17	1	1.00	1.00	1.11	1.00	1.00	1.00	1.05	1.07	1.16	1.06	1.03	1.05
18	0	1.00	1.00	1.00	1.00	1.00	1.00	1.00	1.00	1.13	1.00	1.00	1.00
19	1	1.00	1.00	1.66	1.00	1.00	1.00	1.00	1.00	1.83	1.00	1.00	1.00
20	1	1.00	1.00	1.13	1.00	1.00	1.00	1.00	1.00	1.13	1.00	1.00	1.00
21	1	2.09	2.86	2.09	2.13	2.07	2.09	2.15	2.86	2.15	2.18	2.15	2.15
22	1	1.00	1.00	1.00	1.00	1.00	1.00	1.00	1.00	1.00	1.00	1.00	1.00
23	1	1.44	1.44	1.77	1.44	1.40	1.44	1.45	1.45	1.88	1.45	1.42	1.45
24	1	1.00	1.00	1.00	1.00	1.00	1.00	1.00	1.00	1.00	1.07	1.00	1.00
25	1	1.00	1.00	1.00	1.00	1.00	1.00	1.00	1.00	1.00	1.00	1.00	1.00
26	1	1.08	1.08	1.12	1.08	1.07	1.08	1.11	1.11	1.12	1.11	1.11	1.11

27	1	1.00	1.00	1.00	1.00	1.00	1.00	1.00	1.00	1.00	1.00	1.00	1.00
28	1	1.00	1.00	1.00	1.00	1.00	1.00	1.00	1.00	1.00	1.00	1.00	1.00
29	1	1.00	1.00	1.26	1.00	1.00	1.00	1.01	1.08	1.26	1.04	1.00	1.01
30	1	1.00	1.00	1.00	1.00	1.00	1.00	1.00	1.00	1.00	1.00	1.00	1.00
31	1	1.00	1.00	1.00	1.00	1.00	1.00	1.00	1.07	1.13	1.00	1.00	1.00
32	1	1.00	1.00	1.00	1.00	1.00	1.00	1.00	1.00	1.00	1.00	1.00	1.00
33	1	1.00	1.00	1.00	1.00	1.00	1.00	1.00	1.00	1.29	1.00	1.00	1.00
34	1	1.00	1.00	1.00	1.00	1.00	1.00	1.00	1.00	1.00	1.00	1.00	1.00
35	1	1.00	1.00	1.00	1.00	1.00	1.00	1.00	1.00	1.00	1.00	1.00	1.00
36	1	1.00	1.00	1.00	1.00	1.00	1.00	1.00	1.00	1.00	1.00	1.00	1.00
Average		1.07	1.10	1.17	1.07	1.06	1.07	1.14	1.18	1.25	1.16	1.10	1.14

[1]Reference model built based on initial discussion with two MOH ophthalmologists

DC – centre with day surgery services
