# Supplementary material for: Improving the effectiveness of service delivery in the public healthcare sector: the case of ophthalmology services in Malaysia
Source: BMC Health Serv Res. 2015 Aug 28;15:349. doi: 10.1186/s12913-015-1011-0 (PMC4551382; doi:10.1186/s12913-015-1011-0)
Supplement: Additional file 4: — Scale type, scale and congestion efficiency scores by DMU and index year. (PDF 125 kb) [file 12913_2015_1011_MOESM4_ESM.pdf]

Additional file 4 - Scale type, scale and congestion efficiency score by DMU and index year

	Year 2011				Year 2012		
DMU Index	Scale Efficiency Score	Scale Efficiency Type	Congestion Efficiency Score	DMU Index	Scale Efficiency Score*	Scale Efficiency Type*	Congestion Efficiency Score*
1	1.47	drs	1.00	1	1.81	irs	1.00
2	1.06	drs	1.22	2	1.01	drs	1.25
3	1.01	drs	1.00	3	1.01	drs	1.04
4	1.00	crs	1.00	4	1.00	crs	1.00
5	1.00	crs	1.00	5	1.00	crs	1.00
6	1.38	drs	1.00	6	1.42	drs	1.00
7	1.35	drs	1.00	7	1.94	drs	1.00
8	1.25	drs	1.00	8	1.00	crs	1.00
9	1.02	drs	1.07	9	1.00	crs	1.00
10	1.02	drs	1.00	10	1.00	crs	1.00
11	1.00	crs	1.00	11	1.00	crs	1.00
12	1.04	drs	1.47	12	1.00	drs	1.35
13	1.22	drs	1.23	13	1.14	drs	1.25
14	1.00	crs	1.00	14	1.00	crs	1.00
15	1.00	crs	1.00	15	1.00	crs	1.00
16	1.00	crs	1.00	16	1.00	crs	1.00
17	1.12	irs	1.00	17	1.05	irs	1.00
18	1.00	crs	1.00	18	1.00	crs	1.00
19	1.06	drs	1.13	19	1.00	crs	1.00
20	1.00	drs	1.48	20	1.00	crs	1.00
21	1.09	drs	1.28	21	1.03	drs	2.09
22	1.02	drs	1.00	22	1.00	crs	1.00
23	1.17	drs	1.31	23	1.00	drs	1.44
24	1.00	crs	1.00	24	1.00	crs	1.00
25	1.00	crs	1.00	25	1.00	crs	1.00
26	1.01	drs	1.05	26	1.03	drs	1.08
27	1.24	drs	1.67	27	1.00	crs	1.00
28	1.08	drs	1.09	28	1.00	crs	1.00
29	1.03	drs	1.44	29	1.01	drs	1.00
30	1.00	crs	1.00	30	1.00	crs	1.00
31	1.00	crs	1.00	31	1.00	crs	1.00
32	1.00	crs	1.00	32	1.00	crs	1.00
33	1.00	crs	1.00	33	1.00	crs	1.00
34	1.00	crs	1.00	34	1.00	crs	1.00
35	1.00	crs	1.00	35	1.00	crs	1.00
36	1.00	crs	1.00	36	1.00	crs	1.00
	1.07		1.07		1.07		1.05

*Congestion efficiency score were calculated using VRS model ; crs = constant return to scale; drs = decreasing return to scale; vrs = variable return to scale
