# Supplementary material for: Improving the effectiveness of service delivery in the public healthcare sector: the case of ophthalmology services in Malaysia
Source: BMC Health Serv Res. 2015 Aug 28;15:349. doi: 10.1186/s12913-015-1011-0 (PMC4551382; doi:10.1186/s12913-015-1011-0)
Supplement: Additional file 5: — VRS technical efficiency scores under standard DEA and bootstrap DEA with their respective rankings. (PDF 48 kb) [file 12913_2015_1011_MOESM5_ESM.pdf]

Additional file 5 - VRS technical efficiency scores under standard DEA and bootstrap DEA with the respective rankings.

The rectangular boxes indicate the agreement between the inefficient DMUs identified by standard DEA (ES < 1) and the low performing DMUs identified by bootstrap DEA.

2011					2012				
DMU ranked by sDEA VRS ES	sDEA VRS ES	DMU ranked by bsDEA VRS ES	bsDEA VRS ES	% Agreement[1]	DMU ranked by sDEA VRS ES	sDEA VRS ES	DMU ranked by bsDEA VRS ES	bsDEA VRS ES	% Agreement[1]
27	1.67	27	1.78	100.0%	21	2.09	21	2.17	85.7%
12	1.47	12	1.57		23	1.44	23	1.5	
20	1.48	20	1.57		12	1.35	12	1.41	
29	1.44	29	1.53		2	1.25	2	1.31	
23	1.31	23	1.38		13	1.25	13	1.31	
21	1.28	21	1.36		26	1.08	26	1.13	
2	1.22	19	1.2		3	1.04	6	1.09	
13	1.23	2	1.29		25	1	32	1.09	
19	1.13	13	1.29		27	1	16	1.09	
28	1.09	28	1.16		28	1	24	1.09	
9	1.07	9	1.14		29	1	34	1.09	
26	1.05	26	1.12		30	1	18	1.09	
36	1	11	1.12		31	1	7	1.09	
10	1	33	1.11		32	1	27	1.09	
11	1	17	1.11		33	1	36	1.09	
14	1	4	1.11		34	1	5	1.09	
15	1	30	1.11		35	1	30	1.09	
16	1	18	1.11		36	1	1	1.09	
17	1	1	1.11		24	1	15	1.09	
18	1	31	1.11		6	1	20	1.08	
22	1	5	1.11		7	1	31	1.08	
24	1	35	1.11		8	1	9	1.08	
25	1	15	1.11		9	1	3	1.08	
30	1	16	1.11		10	1	8	1.08	
31	1	34	1.11		11	1	17	1.08	
32	1	24	1.11		14	1	14	1.08	

33	1	6	1.11	15	1	11	1.08
34	1	36	1.11	16	1	25	1.08
35	1	7	1.11	17	1	19	1.08
5	1	14	1.1	18	1	33	1.08
6	1	22	1.1	19	1	4	1.08
7	1	25	1.1	20	1	22	1.08
8	1	10	1.1	22	1	35	1.08
3	1	8	1.09	5	1	28	1.07
4	1	32	1.09	4	1	29	1.05
1	1	3	1.08	1	1	10	1.04

[1] % Agreement - the proportion of DMU ranked by bsDEA that agrees with the inefficient DMU (ES < 1)
DMU = decision making unit, i.e. 36 ophthalmology centres
VRS = variable return to scale model
sDEA = standard DEA
bsDEA = bootstrap DEA
